# Single-cell ATAC-seq: strength in numbers

**DOI:** 10.1186/s13059-015-0737-7

**Published:** 2015-08-21

**Authors:** Sebastian Pott, Jason D. Lieb

**Affiliations:** Department of Human Genetics, The University of Chicago, 920 E. 58th Street, Chicago, IL 60637 USA

## Abstract

Single-cell ATAC-seq detects open chromatin in individual cells. Currently data are sparse, but combining information from many single cells can identify determinants of cell-to-cell chromatin variation.

## From populations to single cells, ATAC-seq detects open chromatin

ATAC-seq (assay for transposase-accessible chromatin) identifies regions of open chromatin using a hyperactive prokaryotic Tn*5*-transposase, which preferentially inserts into accessible chromatin and tags the sites with sequencing adaptors [1]. The protocol is straightforward and robust and has become widely popular. Up to this point, ATAC-seq and other methods for the identification of open chromatin have required large pools of cells [1, 2], meaning that the data collected reflect cumulative accessibility across all cells in the pool. Now, independent studies from the Shendure and Greenleaf laboratories have modified the ATAC-seq protocol for application to single cells (scATAC-seq) [3, 4]. These studies provide a first look at cell-to-cell variability in chromatin organization by gathering data on hundreds [3] or thousands [4] of single cells in parallel.

## How were the single-cell chromatin measurements obtained?

**Fig. 1:**
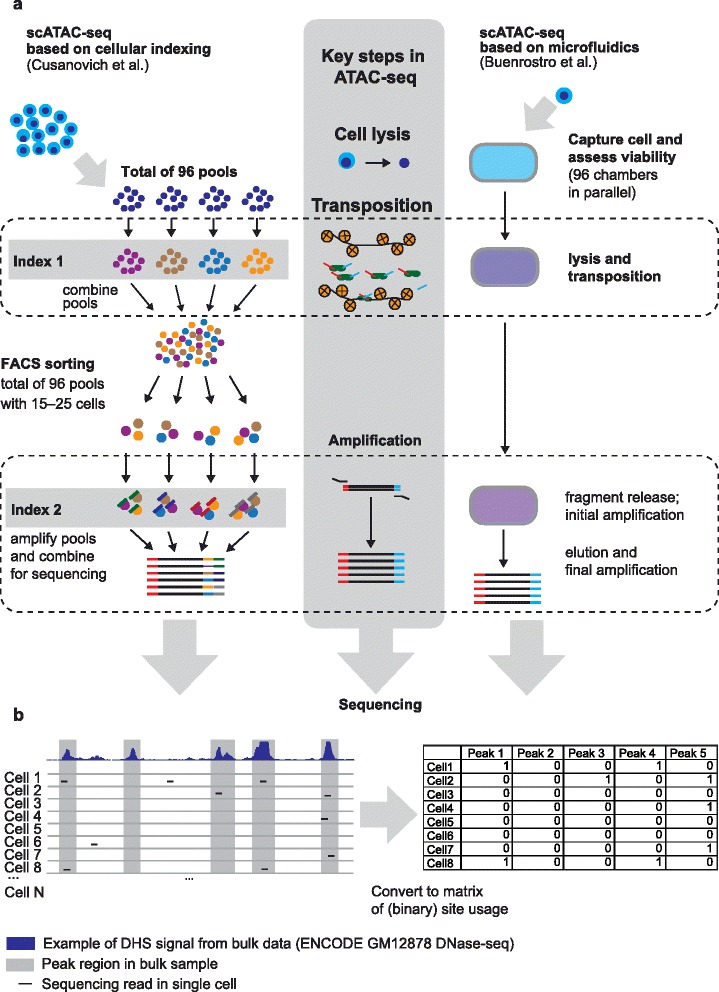
**a** Single-cell assay for transposase-accessible chromatin (*scATAC-seq*) methods. Key steps of the ATAC-seq protocol are shown in the *middle column*. The *left panel* summarizes the scATAC-seq protocol based on cellular indexing established by Cusanovich et al. [4]. The *right panel* illustrates the microfluidic-based protocol introduced by Buenrostro et al. [3]. *FACS* fluorescence-activated cell sorting. **b** ATAC-seq from single cells is sparse and near binary. The properties of chromatin accessibility data from pooled cells (DNase hypersensitive sites (*DHS*) data from GM12878 cells on the top in *dark blue*) and a cartoon representation of how reads from scATAC-seq data might be distributed throughout the same genomic region

Two very different approaches were used: one relied on physical isolation of single cells [3], and the other avoided single-cell reaction volumes by using a two-step combinatorial indexing strategy [4] (Fig. 1a, left panel). In the indexing scheme, Cusanovich et al. [4] lysed cells, and 2500 nuclei were placed into each well of a 96-well plate. Transposases loaded with unique adaptors were added to each well, creating 96 pools of approximately 2500 nuclei, each pool with distinct barcodes. Nuclei from all of the transposition reactions were mixed, and using a fluorescence-activated cell sorter (FACS) 15–25 nuclei were deposited into each well of a second 96-well plate. Nuclei in each well of this second plate were lysed, and the DNA was amplified using a primer containing a second barcode. The low number of nuclei per well ensured that about 90 % of the resulting barcode combinations were unique to a single cell. This combinatorial indexing strategy enabled the recovery of 500–1500 cells with unique tags per experiment. Overall Cusanovich et al. obtained scATAC-seq data from over 15,000 individual cells from mixtures of GM12878 lymphoblastoid cells with HEK293, HL-60, or mouse Patski cells. The number of reads associated with any single cell was very low, varying from 500 to about 70,000 with a median of fewer than 3000 reads per cell.

Buenrostro et al. [3] used a programmable microfluidic device (C1, Fluidigm) to isolate single cells and perform ATAC-seq on them in nanoliter reaction chambers (Fig. 1a, right panel). Each nanochamber was analyzed under a microscope to ensure that a single viable cell had been captured. This approach is simple and has the significant advantage of a carefully monitored reaction environment for each individual cell, although the throughput was limited to processing 96 cells in parallel. Buenrostro et al. sampled 1632 cells from eight different cell lines, including GM12878, K562, and H1 cells, and obtained an average of 73,000 reads per cell, about 20 times the number of reads per cell obtained using the barcoding strategy.

## Does scATAC-seq capture validated open chromatin signal from single cells?

It is important to assess (1) whether the methods generate interpretable open chromatin information, and (2) whether the data are actually from single cells. Regarding (1), both studies show that the distribution of fragment sizes was characteristic of nucleosome-based inhibition of transposase insertion. In addition, both studies showed good overall correlation with chromatin accessibility from traditional bulk datasets, particularly from the lymphoblastoid cell line GM12878 and myeloid leukemia K562 cells [3, 4]. Further, aggregated data from 254 individual GM12878 cells yielded an accessibility pattern highly similar to the pattern produced by population-based ATAC-seq and DNase-seq approaches [3]. Thus, scATAC-seq data capture characteristic features of open chromatin.

Whether the data are actually from single cells is simple to assess in the case of the microfluidic approach because the number of cells captured in each chamber is verified visually [3]. In contrast, combinatorial cellular indexing relies on the presumed low probability of two cells carrying the identical barcode. To test this presumption, the researchers mixed human and mouse cells and found that reads associated with a single barcode map almost exclusively to either the human or mouse genome (the “collision” rate was 11 %) [4]. Therefore, there is strong evidence that both methods generate interpretable chromatin data from single cells.

## Single-cell chromatin data require a new analytic framework to account for fundamental differences from population-based data

Open chromatin data derived from populations of cells exhibit a wide range of signal intensity across the genome. But at the level of single cells the signal is binary, comprising 0 or 1 independent reads in a region (counts of 2, 3, or more, corresponding to multiple insertions in a single region or to other alleles of a locus, are theoretically possible but would be rare). Due to the sparse nature of the data it is therefore impossible to tell if a region that went unobserved in a single cell but was observed in bulk samples is in fact inaccessible in that cell, or was simply missed by the transposase, or was lost in the amplification process. This limitation can be overcome for some purposes by sampling many cells in parallel or by analyzing sets of insertion sites with shared features. This type of aggregation allows one to summarize the binary observations in single cells as frequencies observed on the level of many cells or many sites, respectively. Both studies used this approach, and developed analytical frameworks that relied on chromatin accessibility information from pooled cells to interpret their scATAC-seq data (Fig. 1b).

Cusanovich et al. compared the reads from each cell to DNase hypersensitive sites (DHSs) from ENCODE to produce a binary map of chromatin accessibility, annotating each DHS region as “used” or “unused” based on the overlap. They compared these binary maps among all pairwise combinations of cells to determine similarities and differences among them. This information was sufficient to deconvolute mixtures of two cell lines into their cell types of origin. Further analysis focused on clusters of regions with coordinated chromatin accessibility within a cell type, identifying subpopulations of GM12878 cells [4].

The analysis by Buenrostro et al. focused on identifying factors associated with cell-to-cell variability of chromatin accessibility. They reasoned that trans-factors might influence variability in chromatin accessibility — for example, by binding to accessible chromatin. They first obtained regions of open chromatin using aggregate single-cell data and then grouped these regions into ensembles of sites that shared genomic features based on ChIP-seq data, DNA sequence motifs, or domains with similar replication timing. Using the signal across all cells, they then calculated a “variability score” for each factor to measure the associations of hundreds of trans-factors with cell-to-cell variability of chromatin accessibility.

## What do data from single cells tell us that population-based approaches do not?

The data from these studies reliably separated cells based on their cell types, uncovered sources of cell-to-cell variability, and demonstrated a link between chromatin organization and cell-to-cell variation, all things that population-based approaches could not have told us. Specifically, Buenrostro et al. found that high cell-to-cell variability in chromatin accessibility was associated with binding of specific transcription factors and with replication timing. In K562 cells, GATA1 and GATA2, two central regulators of the erythroid lineage, were both strongly associated with high cell-to-cell variation. Some trans-factors acted synergistically to amplify variation, while others, including CTCF, seemed to suppress variability. Trans-factors associated with high cell-to-cell variability tended to be cell type-specific. For example, Buenrostro et al. found that NFκB binding was associated with cell-to-cell variability in GM12878 cells, but not in K562 and embryonic stem cells. Similarly, Cusanovich et al. found that NFκB binding regions were highly associated with accessible regions that drove the separation of 4118 GM128787 cells into four clusters. Further, the studies demonstrated that cell-to-cell variability is a dynamic phenomenon that can be tuned through extracellular signaling. This was shown by pharmacological perturbation; for example, treatment with tumor necrosis factor-α led to a marked increase in variability of NFκB-associated regions in GM12878 cells, and cell cycle inhibition in K562 cells led to a reduction in chromatin variability of regions associated with specific replication timing. Finally, a connection between chromatin accessibility in cis and chromosome organization was suggested by the finding that groups of adjacent peaks whose deviation correlates with other groups of adjacent peaks across cells mapped to interaction domains previously identified using Hi-C.

## The promise and limitations of probing chromatin in single cells

These studies are important technical advances that demonstrate the promise of scATAC-seq. As one example, the method could be used to characterize cell-to-cell heterogeneity in tumor samples, and may even provide a way to map chromatin accessibility in all individual cells of an organism — for example, during development. One major limitation to current scATAC-seq approaches is that they capture only a tiny subset of the open chromatin sites in single cells, and it seems unlikely that comprehensive coverage can be achieved in the near term. However, higher per-cell coverage would allow new questions to be answered. For example, it is not clear how many open chromatin regions exist in a single cell, or how chromatin accessibility differs between the two alleles in an individual cell. A more comprehensive map would also provide a better understanding of the interplay and co-regulation of multiple regulatory elements associated with a single gene. Recently, single-cell RNA-seq studies were dramatically parallelized by processing thousands of individual cells in miniscule droplets [5]. If a similar approach can be applied to scATAC-seq, one may be able to combine the advantages of the combinatorial indexing used by Cusanovich et al. with the higher per-cell coverage achieved by the microfluidic approach of Buenrostro et al.

## Abbreviations

ATAC-seq: Assay for transposase-accessible chromatin
DHS: DNase hypersensitive site
scATAC-seq: single-cell ATAC-seq
